# Blood biomarkers and neurodegeneration in individuals exposed to repetitive head impacts

**DOI:** 10.1186/s13195-023-01310-w

**Published:** 2023-10-12

**Authors:** Charles Bernick, Guogen Shan, Aaron Ritter, Nicholas J. Ashton, Kaj Blennow, Juan Lantero-Rodriguez, Anniina Snellman, Henrik Zetterberg

**Affiliations:** 1 Neurological Institute, Cleveland Clinic, Las Vegas, NV USA; 2 Department of Biostatistics, University of Florida, Gainesville, FL USA; 3 Department of Psychiatry and Neurochemistry, Institute of Neuroscience and Physiology, the Sahlgrenska Academy at the University of Gothenburg, Mölndal, Sweden; 4 Clinical Neurochemistry Laboratory, Sahlgrenska University Hospital, Mölndal, Sweden; 5 Department of Neurodegenerative Disease, UCL Institute of Neurology, Queen Square, London, UK; 6 UK Dementia Research Institute at UCL, London, UK; 7 Hong Kong Center for Neurodegenerative Diseases, Clear Water Bay, Hong Kong, China; 8 Wisconsin Alzheimer’s Disease Research Center, School of Medicine and Public Health, University of Wisconsin, University of Wisconsin-Madison, Madison, WI USA

**Keywords:** Biomarkers, Chronic traumatic encephalopathy, Traumatic brain injury, Neurodegeneration

## Abstract

**Background:**

It is unknown if fluid biomarkers reflective of brain pathologies are useful in detecting and following a neurodegenerative process in individuals exposed to repetitive head impacts. This study explores the relationship between blood biomarkers and longitudinal change in cognitive function and regional brain volumes in a cohort of professional fighters.

**Methods:**

Participants are drawn from a convenience sample of active and retired professional boxers and Mixed Martial Arts fighters and a control group with no prior exposure to head impacts. 3 T MRI brain imaging, plasma samples, and computerized cognitive testing were obtained at baseline and, for a subset, annually. MRI regional volumes were extracted, along with plasma levels of neurofilament light chain (NfL), glial fibrillary acidic protein (GFAP), p-tau231, and N-terminal tau (NTA). Statistical analyses were performed to assess the relationship between plasma levels and regional brain volumes and cognitive performance at baseline and longitudinally.

**Results:**

One hundred forty active boxers (mean age: 31 with standard deviation (SD) of 8), 211 active MMA (mean age of 30 with SD of 5), 69 retired boxers (mean age 49 with SD of 9), and 52 control participants (mean age 36 with SD of 12) were included in the analyses. Baseline GFAP levels were highest in the retired boxers (retired boxers v. active MMA: *p* = 0.0191), whereas active boxers had higher levels of NfL (active boxers v. MMA: *p* = 0.047). GFAP showed an increase longitudinally in retired boxers that was associated with decreasing volumes of multiple cortical and subcortical structures (e.g., hippocampus: *B* =  − 1.25, 95% CI, − 1.65 to − 0.85) and increase in lateral ventricle size (*B* = 1.75, 95% CI, 1.46 to 2.04). Furthermore, performance on cognitive domains including memory, processing speed, psychomotor speed, and reaction time declined over time with increasing GFAP (e.g., processing speed: *B* =  − 0.04, 95% CI, − 0.07 to − 0.02; reaction time: *B* = 0.52, 95% CI, 0.28 to 0.76). Among active fighters, increasing levels of GFAP were correlated with lower thalamic (*B* =  − 1.42, 95% CI, − 2.34 to -0.49) and corpus callosum volumes, along with worsening scores on psychomotor speed (*B* = 0.14, 95% CI, 0.01 to 0.27).

**Conclusion:**

Longitudinal plasma GFAP levels may have a role in identifying individuals exposed to repetitive head impacts who are at risk of showing progressive regional atrophy and cognitive decline.

**Supplementary Information:**

The online version contains supplementary material available at 10.1186/s13195-023-01310-w.

## Background

Extensive exposure to repetitive head impacts (RHI) increases the risk of long-term neurological impairment including chronic traumatic encephalopathy (CTE) [1]. However, not everyone exposed to RHI will experience neurological decline and among those who do, the onset of symptoms may be many years or decades after exposures [2]. Because of this, the ability to predict or track changes occurring in the brain in either those still, or previously, exposed to RHI has numerous implications both on a personal and research basis. To this end, there is a great interest in identifying biomarkers that could be used to detect the development of a neurodegenerative process and/or follow progression over time [3, 4].

Among biomarkers under investigation, most are either imaging or fluid (blood or CSF) based. As has been seen in the field of Alzheimer’s disease, blood biomarkers have the potential to be used to screen or support a clinical diagnosis or become an outcome measure in clinical therapeutic trials [5, 6]. With the availability of sensitive blood-based assays, a number of candidates have been studied in traumatic brain injury including neurofilament light chain (NfL), glial fibrillary astrocytic protein (GFAP), and various species of tau [7, 8]. However, much of the prior longitudinal research with these measures has had relatively short follow up, limited outcome measures, or studied groups exposed to single traumatic brain injuries of varying severities [9–11].

The Professional Athletes Brain Health Study is a longitudinal cohort study of both active and retired professional fighters. Utilizing this well characterized cohort of individuals exposed to RHI and followed over time, we chose four plasma biomarkers to examine: GFAP (a marker of astrocytic injury or activation), NfL (a marker of neuroaxonal injury and degeneration), p-tau231 (a marker of tau phosphorylation), or N-terminal tau ([NTA tau] a novel plasma biomarker specific for AD pathology). The primary aims of the study were to determine (1) whether baseline biomarker levels were correlated with cognitive performance or MRI regional volume in individuals exposed to RHI, (2) whether baseline biomarker levels predict subsequent change over time in cognition or MRI regional volumes, and (3) does longitudinal trajectory of these markers correlate with change over time in the outcomes?

## Methods

### Cohort

The Professional Athletes Brain Health study (PABHS) is composed of active and retired professional fighters (boxers and mixed martial artists), along with controls. Active fighters were required to have at least 1 professional fight within 2 years of enrollment and be training with the intent to compete. Retired fighters were included if they had been boxers or mixed martial artists, had a minimum of 10 professional fights, had no sanctioned fights for at least 2 years, and did not intend to return to competition. Control subjects were recruited from outreach efforts in the community and could not have any prior history of neurological disorders, head trauma, military service, or participation at a high school level or higher in a combat sport or a sport in which head trauma can be anticipated to occur, such as football, wrestling, hockey, rugby, soccer, or rodeo. Enrollment in the PABHS began in 2011 and has been continuous since then. Each participant is seen on an annual basis and, for active fighters, not sooner than 45 days from a sanctioned fight to reduce the potential acute effects of head impacts sustained in competition. Because of a variety of reasons (training and competition schedule, travel issues, other obligations), participants who missed a study visit were allowed to remain in the study with the next study visit conducted as soon as they were available. We consider the “baseline” blood levels as the ones that were drawn at the first study visit. Data for this study were collected between 2011 and 2018.

### Procedures

At each visit, blood sampling is obtained, along with a battery of other tests including MRI brain imaging, computerized cognitive testing, and exposure information. The PABHS was approved by the Cleveland Clinic Institutional Review Board, and written informed consent was obtained from all participants. Methods of recruitment and study procedures have been described previously [12].

Cognitive function was assessed by a computer-based battery consisting of four subtests of the CNS Vital Signs (CNS Vital Signs, North Carolina) including verbal memory, symbol digit coding, Stoop, and a finger tapping test. CNS Vital Signs offers robust and reliable measurements of cognition which are computerized but are supervised by a technician [13]. Results from these tests are used to make up scores in various clinical domains: verbal memory, processing speed, psychomotor speed, and reaction time. Raw scores were used in the analyses.

A high-resolution T1-weighted anatomical MRI was obtained on a 3 T MRI scanner (Siemens Verio from April 2011 through October 2015 and Siemens Skyra from December 2016 to the present) with a 32-channel head coil to acquire structural three-dimensional T1-weighted magnetization prepared rapid acquisition gradient echo images (repetition time msec/echo time msec, 2300/2.98; resolution, 1 X 1 X1.2 mm^3^. Volumes of the hippocampus, amygdala, superior temporal, various frontal regions, anterior cingulate, and total gray matter and subcortical grey matter including thalamus, caudate, and putamen, along with corpus callosum and total white matter volume, were calculated using the automated full brain segmentation process in the Freesurfer 6.0 software. These regions have been shown in pathological series and our prior work to be affected in those with extensive RHI [14, 15]. The volumes of each structure were measured in both hemispheres separately and an average volume calculated for structures that have bilateral representation. The regional volumes were adjusted for total intracranial volume (TIV) by adding TIV as a covariate. A quality control step was performed using the FreeSurfer’s quality analysis tools (https://surfer.nmr.mgh.harvard.edu/fswiki/QATools) to guarantee only data with high-quality cortical reconstruction from FreeSurfer were included in the analyses.

The blood samples were collected in EDTA tubes and centrifuged at 3200 rpm for 10 min to separate plasma from blood cells. The supernatant was aliquoted in 2 ml portions that were immediately frozen and stored at – 80° pending analysis. For all measured biomarkers (commercially available or in-house developed), plasma samples were allowed to thaw at room temperature for 45 min, after which they were vortexed (at 400 rpms for 30 s) and centrifuged (4000 g for 10 min). Internal quality controls (iQC) were included on each plate before and after the analyzed samples to determine inter- and intra-assay variability (intra- and inter-assay variation was < 15% for all biomarkers). All blood biomarkers were measured using a Simoa HD-X platform (Quanterix, Billerica, MA, USA) at the Clinical Neurochemistry Laboratory, Sahlgrenska University Hospital, Mölndal, Sweden. NfL and GFAP were measured using commercially available Simoa kits (NF-light™ #103,186 and GFAP #102,336, Quanterix, Billerica, MA, USA) following manufacturer specifications. In-house developed plasma p-tau231 and NTA tau are measured following published protocols [16, 17]. In brief, plasma p-tau231 assay is comprised by a mouse monoclonal antibody selective against phosphorylated tau at threonine 231 (ADx253, ADx Neuroscience) and a biotinylated mouse monoclonal antibody with epitope at aa 6–18 (Tau12, #806,501, Biolegend). Recombinant full-length Tau-441 phosphorylated in vitro by glycogen synthase kinase 3β (#T08-50FN, SignalChem) was used as the calibrator. The plasma NTA assay targets N-terminal tau fragments using two mouse monoclonal antibodies, with epitopes at aa 6–18 (Tau12, #806,501, Biolegend) and 194–198 (HT7, #MN1000, Thermo Scientific). Recombinant non-phosphorylated full-length Tau-441 (#T08-54N, SignalChem) was used as the calibrator.

Genotyping of apolipoprotein E (APOE) alleles was performed using real-time PCR restriction fragment length polymorphism analysis. Briefly, genomic DNA was collected from blood DNA extracted using Qiamp DNA blood maxi kit (Qiagen), and APOE genotyping was performed using Applied Biosystems TaqMan SNP Genotyping Assay.

STROBE reporting guideline was adhered to in preparing this manuscript.

### Statistical analysis

The cohort was divided into four groups for analyses: active boxers, active MMA fighters, retired boxers, and controls. We chose to divide the active fighters by their fighting discipline because of prior findings from the PABHS that indicate active boxers show lower regional gray matter volumes and lower scores on cognitive tests after adjusting for number of professional fights and other factors compared to the active MMA fighters [15, 18]. We examined only retired boxers because we did not have enough retired MMA fighters in the cohort to analyze separately.

For the comparison of demographic data between the four groups in this study cohort, Kruskal–Wallis test was used for continuous outcomes (e.g., age, number of fights), and the chi-squared test was used for categorical outcomes (e.g., race). For continuous outcomes, we reported the interquartile range (IQR) values with the mean value. For *APOE* ε4 positivity, Fisher’s exact test was used for comparing the four groups [19].

**Fig. 1:**
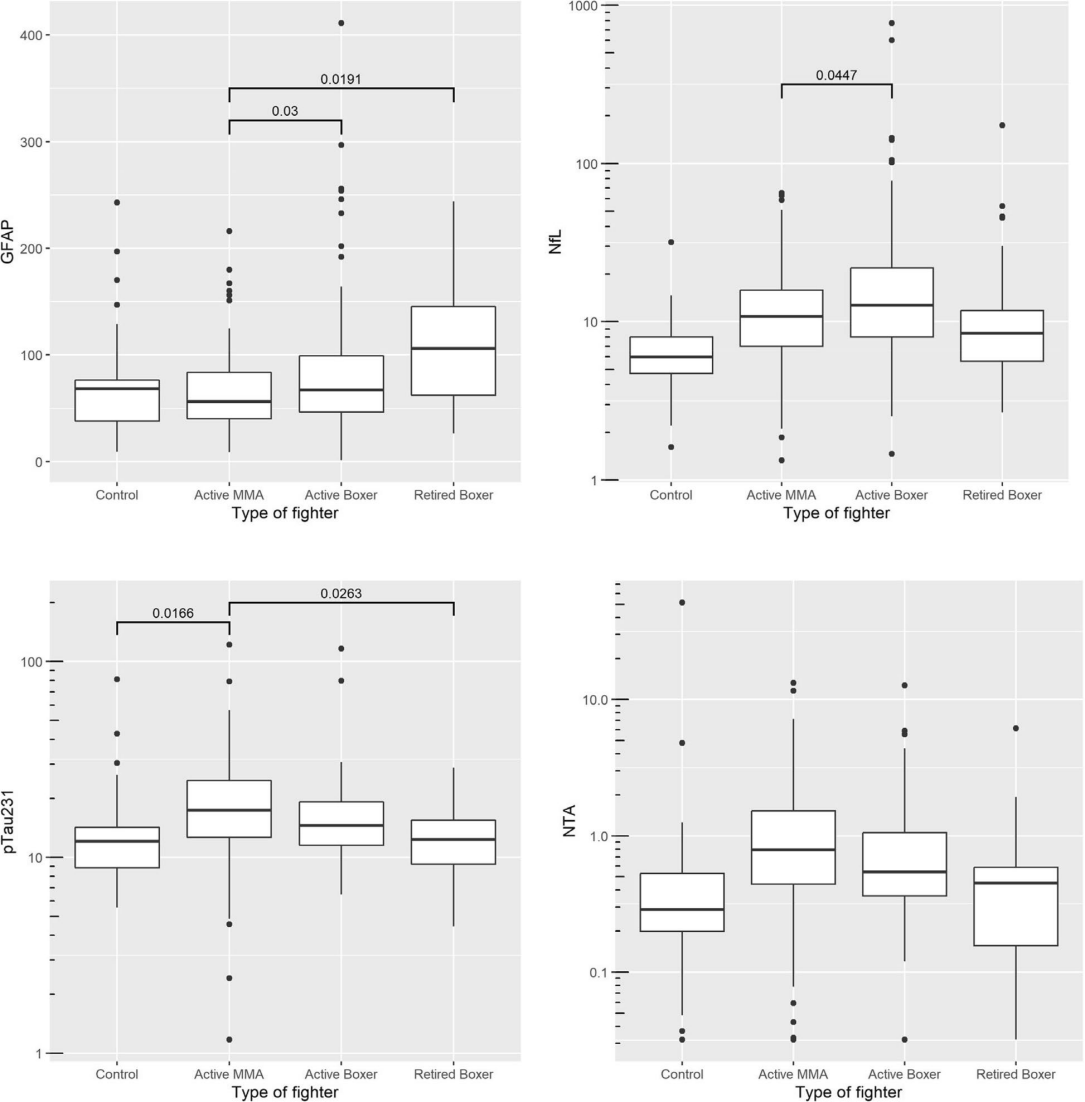
Baseline levels of GFAP, NfL, p-Tau 231, and NTA Tau in control subjects, active MMA fighters, active boxers, and retired boxers (measured in pg/mL)

We used linear regression models to compare the baseline blood biomarkers (GFAP, NfL, p-tau231, NTA) between the four groups after controlling for age, gender, race, education years, and number of fights (Fig. 1). Race was included as a covariate because of recent reports from the Alzheimer’s disease literature that suggest some fluid biomarkers may vary by race [20, 21]. In the PABHS cohort, race was determined by self-report and included White, African American, Asian, Pacific Islander, and American Indian/Native Alaskan. Those who did not designate a race were placed into the category of *other*. We chose to adjust for number of fights as previous findings from the PABHS indicate that this exposure measure itself predicts both cognitive and MRI volumetric outcomes [22]. In Fig. 1, the reported *p*-values are the ones after the multiple testing correction by using the Tukey’s approach.

For the association between blood biomarkers at baseline and cognitive performance or MRI regional volume at baseline, linear regression models were performed with the covariates: age, gender, race, education years, and number of fights. Though we would have preferred to compare our fighter groups to the controls, we felt we did not have enough controls to match either the younger active fighter groups or the older retired boxer group. Furthermore, plasma levels of all the biomarkers we studied are known to be influenced by age. Thus, we performed within group analysis. When MRI regional volumes are the outcome of interest, another two covariates were added to the statistical model: scanner type and the total intracranial volume (TIV). These two covariates were also added in the following statistical models for repeated MRI regional volumes.

Participants were included in longitudinal analyses if they had two or more visits including blood biomarker, MRI, and cognitive data. Linear mixed effect models were used to assess the relationship between the longitudinal cognitive performance or MRI regional volume and each longitudinal blood biomarker data. As above, each group was analyzed separately. The outcomes are MRI regional volumes or cognitive measures. The fixed effects are blood biomarker, group, and their interaction, age, gender, race, education years, and number of fights. The fixed effect group is a categorical variable with 4 separate arms. The other two covariates, scanner type and the total intracranial volume, were added in the models for repeated MRI regional volumes. The correlation for outcome from the same participant is assumed to be the compound symmetry structure. We also ran linear mixed models to evaluate the relationship between baseline blood biomarker level and the longitudinal cognitive performance or MRI regional volume. The same fixed effects were included in the model.

We checked the statistical model assumptions by virtually inspecting the following plots: residual VS fitted value plot, Q-Q plot. The statistical software SAS was used in the analyses, and software R was used for some plots. All the tests are two-sided with the significance level of 0.05.

## Results

The study cohort was primarily made up of active boxers (*n* = 140) and MMA (*n* = 211) fighters with a smaller number of retired boxers (*n* = 69) and controls (*n* = 52). Demographic and clinical data are presented in Table 1. We also ran post-hoc tests using the Bonferroni method for multiple comparisons. There was a group difference in age except between the active boxers and the active MMA. Education years and number fights were different between fighter groups in the post-hoc comparisons.

**Table 1:** Demographic table for baseline data

	Active boxer	Retired boxer	Active MMA	Control	*p*-value
*N*	140	69	211	52	
Age in years (IQR)	31.03 (26–35)	48.75 (43–54)	30 (27–33)	35.62 (26–44)	< 0.0001
Education years (IQR)	13.06 (12–14)	12.74 (12–14)	13.69 (12–16)	14.96 (12.5–16)	< 0.0001
Number of fights (IQR)	13.46 (3–22)	38.41 (26–50)	12.95 (3–19)	0	< 0.0001
Years of fighting (IQR)	4.44 (1–8)	10.7 (9–15)	3.49 (1–8)	0	< 0.0001
ApoE e4 positivity	35 (31.25%)	15 (24.19%)	42 (23.73%)	15 (31.91%)	0.0014
Male	130 (92.86%)	65 (94.20%)	178 (84.36%)	39 (75.00%)	
Race					< 0.0001
African American	48 (34.29%)	28 (40.58%)	25 (11.85%)	3 (5.77%)	
White	42 (30.00%)	30 (43.48%)	122 (57.82%)	32 (61.54%)	
American Indian/native	0	0	4 (1.90%)	0	
Asian	5 (3.57%)	0	7 (3.32%)	6 (11.54%)	
PI	3 (2.14%)	0	10 (4.74%)	0	
Multiracial	7 (5.00%)	3 (4.35%)	14 (6.64%)	3 (5.77%)	
Others	35 (25.00%)	9 (13.04%)	31 (14.69%)	8 (15.38%)	

The number of participants in the longitudinal analyses included 52 active boxers, 55 retired boxers, 103 active MMA, and 27 controls. This longitudinal subgroup had average years of follow up ranging from 2.73 (SD = 1.52, IQR 1.5–4) in the retired boxers to 3.35 (SD = 1.44, IQR 2–4) for active boxers, 3.09 (SD = 1.57, IQR 2–4) for active MMA, and 2.11 (SD 1.01, IQR 1–3) for controls.

### Baseline levels between groups

Baseline GFAP levels were significantly increased in both retired boxers (mean = 108, SD = 56) and active boxers (mean = 81, SD = 59) compared with active MMA fighters (mean = 65, SD = 35) [retired boxer v. active MMA: *p* = 0.019, active boxer v. active MMA: *p* = 0.03]. NfL increased in active boxers (mean = 30.28, SD = 83.85) as compared to the active MMA (mean = 13.85, SD = 10.95), with *p* = 0.0447. Plasma p-tau231 was increased in active MMA fighters compared with retired boxers (*p* = 0.026) and control (*p* = 0.0166). Plasma NTA baseline levels were not significantly different across groups (Fig. 1).

After removing two outliers with NfL values above 500, the mean NfL difference between active MMA and active boxers was reduced, but the standard error of the mean difference was reduced even more due to smaller variance. For that reason, the adjusted *p*-value for comparing active MMA and active boxers was 0.006. In addition, the difference between active boxers and controls became statistically significant with the adjusted *p*-value of 0.0002.

### Baseline levels and volumetric/cognitive outcomes

Cross-sectional analysis within groups revealed relationships between higher levels of GFAP and lower volumes in various gray and white matter regions and higher ventricular volumes primarily in the retired boxers (Table 2). The structures effect most in the retired boxers included thalamus (*B* =  − 4.12, 95% CI, − 6.44 to − 1.81) hippocampus (*B* =  − 1.90, 95% CI, − 3.46 to − 0.34), inferior lateral ventricle (*B* = 1.46, 95% CI, 0.15 to 2.76), cerebral white matter (*B* =  − 40.72, 95% CI, − 70.4 to − 11.1), and total gray matter (*B* =  − 7.92, 95% CI, − 11.91 to − 3.92). In addition, for retired boxers, lower scores on processing speed (*B* =  − 0.25, 95% CI, − 0.38 to − 0.11) were associated with higher levels of GFAP (Additional file 1: Fig. S3). Among the active boxers, higher GFAP levels correlated with lower volumes in the thalamus and larger volumes of the lateral ventricles. Within-group assessment of NfL showed that higher baseline levels were associated with lower volumes of the thalamus (*B* =  − 1.68, 95% CI, − 2.77 to − 0.61), hippocampus (*B* =  − 0.84, 95% CI, − 1.56 to − 0.112), anterior cingulate (*B* =  − 0.34, 95% CI, − 0.61 to − 0.06), and subcortical gray matter (*B* =  − 7.90, 95% CI, − 14.3 to − 1.47) in the active boxers. On the other hand, no consistent relationships were seen with the volumetric measures and levels of ptau 231 and NTA in any of the groups.

**Table 2:** Relationship between GFAP baseline levels and MRI regional volumes with 95% confidence interval, with no reference. MRI volumes are measured in mm^3^. For cognitive measures, scores are computed from raw score calculations using the data values of individual subtests and are simply the number of correct responses, incorrect responses, and reaction times. Reaction times are in milliseconds. Lower scores of verbal memory, processing speed, and psychomotor speed indicate worse performance; for reaction time, higher scores are worse

	Active boxer	Active MMA	Retired boxer	Control
Brain regions				
Thalamus	− 2.46 (− 4.00, − 0.93)*	− 0.43 (− 2.53, 1.68)	− 4.12 (− 6.44, − 1.81)^***^	0.20 (− 3.27, 3.67)
Caudate	− 0.09 (− 0.93, 1.11)	− 1.10 (− 2.50, 0.30)	− 1.06 (− 2.60, 0.48)	0.35 (− 1.96, 2.65)
Hippocampus	− 0.20 (− 1.23, 0.84)	− 0.76 (− 2.17, 0.66)	− 1.90 (− 3.46, − 0.34)^*^	− 2.02 (− 4.36, 0.31)
Amygdala	0.16 (− 0.33, 0.65)	0.20 (− 0.46, 0.87)	− 0.44 (− 1.18, 0.30)	0.10 (− 1.00, 1.21)
Lateral ventricle	15.41 (1.76, 29.06)^*^	− 2.75 (− 21.48, 15.99)	20.19 (− 0.45, 40.82)	16.47 (− 14.40, 47.35)
Inferior lateral ventricle	0.27 (− 0.59, 1.14)	0.15 (− 1.03, 1.34)	1.46 (0.15, 2.76)^*^	0.07 (− 2.02, 1.88)
Corpus callosum (posterior)	0.11 (− 0.35, 0.56)	0.27 (− 0.90, 0.35)	0.00 (− 0.69, 0.69)	0.25 (− 1.28, 0.79)
White matter	− 63.23 (− 149.20, 22.75)	− 24.52 (− 142.55, 93.50)	− 140.74 (− 270.74, − 10.74)^*^	− 116.32 (− 310.82, 78.18)
Total gray	− 7.62 (− 16.80, 1.57)	− 2.36 (− 14.97, 10.24)	− 21.58 (− 35.46, − 7.69)^**^	2.53 (− 18.24, 23.31)
Cognitive measures				
Processing speed	− 0.05 (− 0.14, 0.04)	− 0.04 (− 0.16, 0.09)	− 0.25 (− 0.38, − 0.11)^***^	− 0.06 (− 0.26, 0.15)
Psychomotor speed	0.06 (− 0.13, 0.00)	0.02 (− 0.11, 0.08)	0.09 (− 0.19, 0.02)	− 0.21 (− 0.36, 0.05)
Verbal memory	− 0.01 (− 0.02, 0.01)	0.01 (− 0.01, 0.03)	− 0.01 (− 0.04, 0.01)	0.00 (− 0.03, 0.03)
Reaction time	0.23 (− 0.11, 0.58)	0.00 (− 0.47, 0.48)	0.47 (− 0.05, 0.99)	− 0.22 (− 1.00, 0.56)
Choice reaction time	0.08 (-0.10, 0.25)	0.21 (− 0.02, 0.44)	0.24 (− 0.02, 0.50)	0.27 (− 0.12, 0.65)

*significant difference at *p* < 0.05 level, **significant difference at *p* < 0.01 level, and ***significant difference at *p* < 0.001 level

Baseline level of GFAP correlated with longitudinal rate of change in both MRI volume and cognitive measures primarily within the retired boxer group. For every unit increase in GFAP at baseline, there was a greater yearly rate of decline in the thalamus (*B* =  − 4.64, 95% CI, − 6.74 to − 2.54), hippocampus (*B* =  − 2.52, 95% CI, − 3.86 to − 1.18), amygdala (*B* =  − 0.67, 95% CI, − 1.33 to − 0.01), and increase in the inferior lateral ventricle (*B* = 2.59, 95% CI, 1.43 to 3.76). There was also a decline in processing speed (*B* =  − 381, 95% CI, − 552 to − 210) and reaction time (*B* = 0.77, 95% CI, 0.31 to 1.23). Baseline measures of the other 3 analytes did not correlate with longitudinal outcomes.

### Longitudinal change and volumetric/cognitive outcomes

**Fig. 2:**
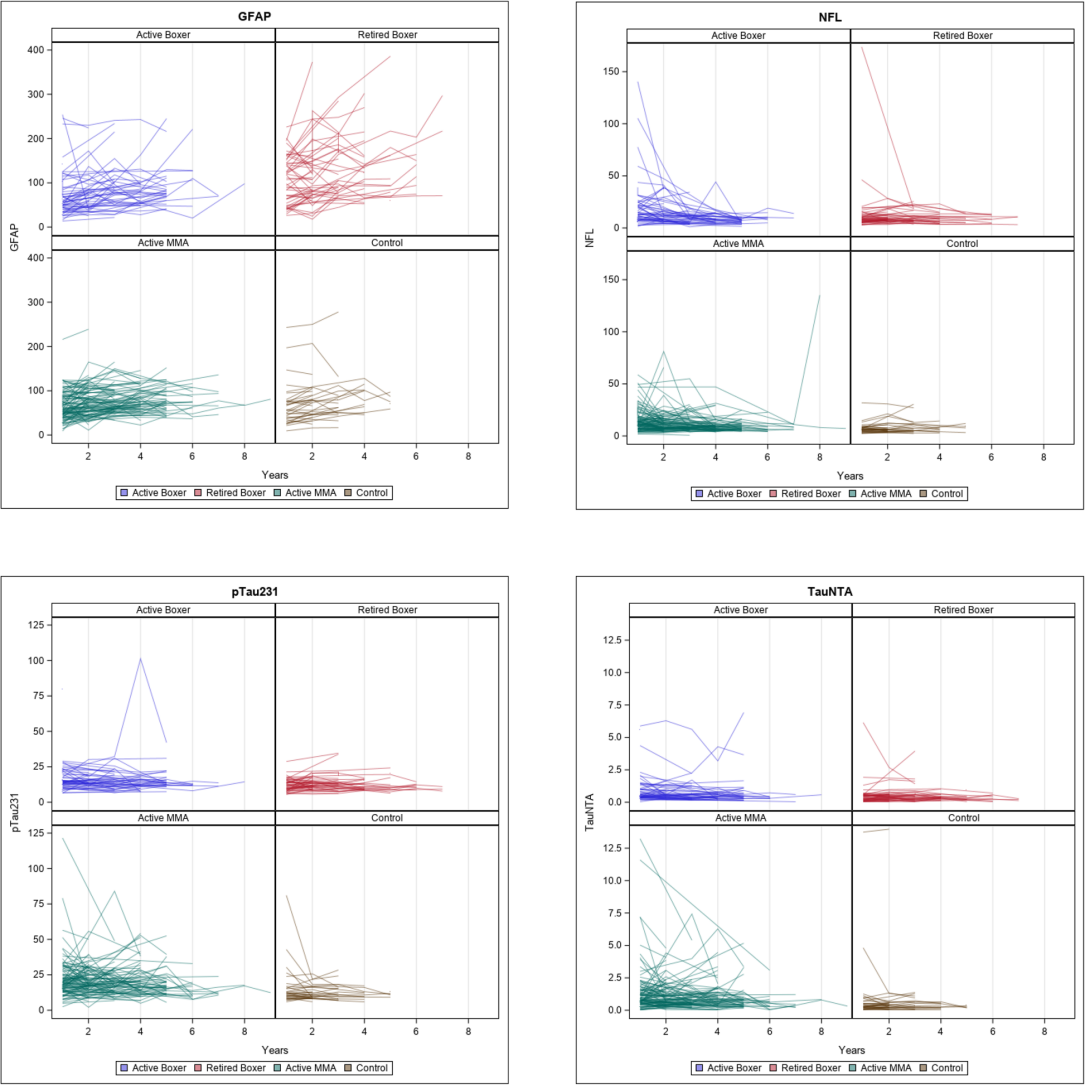
Longitudinal measurement of plasma levels of GFAP, NfL, p-Tau 231, and NTA tau in control subjects, active MMA fighters, active boxers, and retired boxers (measured in pg/mL)

In Fig. 2, we show the longitudinal change of each blood biomarker. Longitudinal repeated-measure models were used to assess the change over time after controlling for the aforementioned covariates. GFAP is the only analyte showing an increase longitudinally. We then further analyzed the longitudinal change of GFAP with the longitudinal change of MRI volumes and cognitive measures in Table 3.

**Table 3:** Relationship between longitudinal GFAP levels and MRI regional volumes with 95% confidence interval, with no reference and cognitive measures. The table shows what a 1 unit/year increase in GFAP level is or is not associated with MRI volumes are in measured in mm^3^. For cognitive measures, scores are computed from raw score calculations using the data values of individual subtests and are simply the number of correct responses, incorrect responses, and reaction times. Reaction times are in milliseconds. Lower scores of verbal memory, processing speed and psychomotor speed indicate worse performance; for reaction time, higher scores are worse

	Active boxer	Active MMA	Retired boxer	Control
Hippocampus (left)	− 0.09 (− 0.31, 0.13)	− 0.04 (− 0.34, 0.26)	− 0.28 (− 0.49, − 0.08)^**^	0.19 (− 0.37, 0.76)
Lateral ventricle	1.13 (0.43, 1.84)^**^	1.27 (0.30, 2.24)^*^	2.19 (1.53, 2.85)^***^	1.73 (− 0.06, 3.52)
Inferior lateral ventricle	− 0.01 (− 0.12, 0.11)	− 0.01 (− 0.18, 0.15)	0.46 (0.35, 0.57)^***^	− 0.10 (− 0.40, 0.20)
Total gray	13.24 (− 3.96, 30.44)	12.65 (− 36.54, 11.25)	32.21 (− 48.20, − 16.22)^***^	36.66 (− 81.66, 8.34)
Psychomotor speed	− 0.008 (− 0.028, 0.012)	− 0.018 (− 0.045, 0.010)	− 0.020 (− 0.038, − 0.002)^***^	0.114 (0.061, 0.167)
Reaction time	0.003 (− 0.111, 0.117)	− 0.016 (− 0.173, 0.141)	0.200 (0.097, 0.303)^***^	− 0.052 (− 0.363, 0.259)
Verbal memory	0.001 (− 0.006, 0.008)	− 0.011 (− 0.020, − 0.002)	− 0.003 (− 0.009,- 0.003)^*^	0.008 (− 0.010, 0.027)

^*^Significant difference at *p* < 0.05 level, **significant difference at *p* < 0.01 level, and ***significant difference at *p* < 0.001 level

Longitudinal increase in GFAP in retired fighters was associated with decreasing volume of multiple cortical and subcortical structures (e.g., hippocampus (left): *B* =  − 0.28, 95% CI, − 0.49 to − 0.0.08) and increase in lateral ventricle size (*B* = 2.19, 95% CI, 1.53 to 2.85) as reported in Table 3. Furthermore, performance on a variety of cognitive domains including memory, psychomotor speed, and reaction time declined over time with increasing GFAP (e.g., psychomotor speed: *B* =  − 0.02, 95% CI, − 0.038 to − 0.002; reaction time: *B* = 0.20, 95% CI, 0.097 to 0.303) (see Additional file 2: Fig. S4). In the active boxer group and the active MMA group, a relationship was seen between increasing levels of GFAP and increase in lateral ventricle size.

## Discussion

In pursuit of biomarkers that may be applicable in detecting progressive neurological change in those exposed to repetitive head impacts, this study evaluated several blood-based measures in a cohort of both active and retired professional fighters, a subset of which had longitudinal imaging and cognitive assessments. In answer to the aims of this study, we found that (1) among the analytes tested, higher levels of GFAP were inversely correlated with regional volumes and cognitive performance in retired boxers. On the other hand, NfL levels were inversely associated with volume measurements at baseline in active boxers. (2) Higher levels of baseline GFAP level were associated with increasing rate of cognitive and MRI volume decline in retired boxers, and (3) increasing levels of GFAP over time were inversely related to rate of decline in retired boxers. The N-terminal tau assay (NTA) and p-tau231 did not seem to have any clear relationships to the outcome measures.

How might the findings of this study translate to clinical or research use, either to screen for (or determine risk of) a neurodegenerative process such as CTE or be employed as a biomarker in clinical trials? To begin with, cross-sectional measurements of any of these biomarkers may not have utility as a diagnostic measure for a condition such as CTE. There was a clear overlap in plasma levels of all biomarkers between our active and retired fighters and control subjects who have not been exposed to RHI. Previous studies of all the blood biomarkers we tested have reported as high or higher absolute levels in other neurodegenerative disease states [23]. What makes it difficult to compare absolute values of these blood constituents between studies are the differences in technical factors such as how the samples are handled and processed and the platforms used for the measurements. In addition, elevated levels of GFAP and NfL (among a number of other plasma biomarkers) have been described following acute exposure to TBI and RHI [4].

On the other hand, following plasma GFAP levels over time may help identify those previously exposed to RHI who are developing a neurodegenerative process such as, but not limited to, CTE. GFAP is an intermediate filament protein that is predominately expressed in astroglial cells and thought to be a marker of astrocyte remodeling and reactivity [24]. Prior studies have shown that GFAP increases with age and may be a marker of Alzheimer’s disease [25]. However, elevations have been reported in other neurodegenerative conditions and GFAP has also demonstrated a biphasic release in blood after acute severe TBI, with initial increase, followed by decreasing levels over the first 6 months and then subsequent increase [7]. In our study, the increasing levels of GFAP in the retired boxers may reflect underlying neuroinflammation and/or astrogliosis manifest by regional volume loss and associated clinical finding of lower performance on cognitive measures. However, in the absence of pathological confirmation, there is no way to truly know what type of pathophysiology the plasma GFAP represents. Furthermore, our findings need to be replicated in other cohorts exposed to RHI.

The other biomarkers we studied may have different applications. Levels of NfL were higher at baseline in active boxers than active MMA fighters and were associated with lower thalamic, hippocampal, and white matter volumes in that group. NfL, an axonal component found primarily in large caliber myelinated subcortical fibers, is one of the more widely studied fluid biomarkers and is thought to be released with axonal injury [26, 27]. Elevated levels of NfL can be seen after acute TBI, with some studies indicating a slow return to baseline extending over the years [7]. Higher levels have also been reported in several neurological diseases such as amyotrophic lateral sclerosis, progressive supranuclear palsy, and Alzheimer’s disease (AD) [28–30]. We propose that the increased levels of NfL in the active boxers may reflect axonal injury from the numerous blows to the head that boxers generally sustain in training and competition; the lower regional volumes perhaps represent the subsequent effects of Wallerian degeneration and neuronal atrophy. The absence of elevated NfL in the retired boxers at baseline and longitudinally differs from what has been described in other neurodegenerative conditions such as AD; though the reason is unclear, it may be that the underlying pathological process associated with RHI in this cohort is more indolent.

The characteristic pathological feature in CTE is the presence of perivascular p-tau deposits at the depths of sulci [10]. Consequently, there has been an interest in evaluating the performance of p-tau measures in CSF or blood as a diagnostic for CTE. Prior work has reported that higher levels of p-tau can be seen soon after TBI which likely represents acute injury [6].

However, the situation is more complicated in trying to use p-tau measures to detect CTE. Tau can be phosphorylated at a variety of sites and there is no agreement on those that are specific or associated with CTE. A recent study that evaluated two p-tau species (p-tau 181, p-tau 217) in a group that included a small number of postmortem verified CTE cases found that these markers were specific for Alzheimer’s disease and not in vivo biomarkers of CTE tau [31].

Among tau phosphorylation sites, those that have been reported with CTE include p-tau 175, 202, 231, and 396 [32, 33]. We chose to study p-tau 231 because it also has been suggested to be specific to the pathology that underpins Alzheimer’s disease, even at the preclinical phase [34]. Similarly, increased blood levels of NTA, which measures non-phosphorylated N-terminal tau species, have been reported to be specific for Alzheimer’ disease and increased in symptomatic patients but little data exists on its performance in other neurological conditions [35]. We did not find that levels of p-tau231 or NTA were related to any of our outcome measures. Our findings suggest that these tau biomarkers are not a marker of a neurodegenerative process related to prior exposure to RHI; the generally flat trajectory of both p-tau 231 and NTA tau over time in the retired boxers also may support the notion that Alzheimer’s disease was not the underlying condition causing the progression seen in MRI volumetrics and cognition in this group.

While this study benefits from having longitudinal biomarker levels and corresponding imaging and cognitive measurements in a large population of individuals with exposure to RHI, there are limitations to discuss. To begin with, because this is not a clinicopathological study, it is impossible to know what process is driving the regional volume changes that were seen in association with some of the fluid biomarkers. In addition, the PABHS does not represent a random sample of professional fighters, and the longitudinal nature of the study raises the possibility that those who participate and are retained in the study may differ in some way from their peers. Another issue regarding the study cohort is our inability to accurately know how much exposure to RHI any individual had. Previous work from the PABHS has indicated that the number of professional fights is a reasonably good surrogate of exposure and is what we employed in adjusting our analyses [22]. There were several differences between fighter groups and controls that limited direct comparison between them and led us to within group analyses. Our control group had the fewest participants and also differed in age than the retired fighters (though there was overlap), being generally younger. Thus, we did not have sufficient numbers of older control participants to compare with the retired fighters. The control group also had a higher percentage of white participants than the fighter groups. The groups also differed in the number of longitudinal plasma samples. The change in MRI scanner during the study period was addressed by adjusting for scanner type in the analyses but could possibly introduce some variability in the regional volume measurements. Plasma biomarker levels could potentially be influenced by BMI which we did not have on all the participants. Finally, there may be technical factors that influence the longitudinal results; many of these samples did undergo multiple freeze/thaw cycles. However, earlier studies have shown that NfL, GFAP, and tau markers are stable even with repetitive freeze/thaw cycles [36].

To conclude, employed as a longitudinal measure, plasma GFAP levels may have a role in identifying individuals who are at increased likelihood of showing progressive regional atrophy and cognitive decline and perhaps could be an outcome measure in clinical trials. On the other hand, NfL measurements seem to be more applicable in those actively exposed to RHI in reflecting neural injury. Further longitudinal studies over greater amounts of time and with different cohorts exposed to RHI are needed to verify our findings.

### Supplementary Information

**Additional file 1: Fig. S3. **Relationship between baseline GFAP (measured in pg/mL) and MRI volume measurements (in mm3) of the thalamus, hippocampus, and total gray matter, along with processing speed (computed from number of correct responses on Symbol Digit Coding test minus errors)

**Additional file 2: Fig. S4. **Longitudinal measurement of reaction time ((Stroop Test Complex Reaction Time Correct + Stroop Reaction Time Correct)/2) and psychomotor speed [Finger Tap Test (FTT) Right Taps Average + FTT Left Taps Average + SDC Correct Responses) in active MMA fighters, active boxers, retired boxers, and control subjects)

## Data Availability

The datasets used for the current study are available from the corresponding author on reasonable request.
