# Supplementary material for: Blood biomarkers and neurodegeneration in individuals exposed to repetitive head impacts
Source: Alzheimers Res Ther. 2023 Oct 12;15:173. doi: 10.1186/s13195-023-01310-w (PMC10571311; doi:10.1186/s13195-023-01310-w)
Supplement: Supplementary file 1 — Additional file 1: Fig. S3. Relationship between baseline GFAP (measured in pg/mL) and MRI volume measurements (in mm3) of the thalamus, hippocampus, and total gray matter, along with processing speed (computed from number of correct responses on Symbol Digit Coding test minus errors) [file 13195_2023_1310_MOESM1_ESM.pdf]

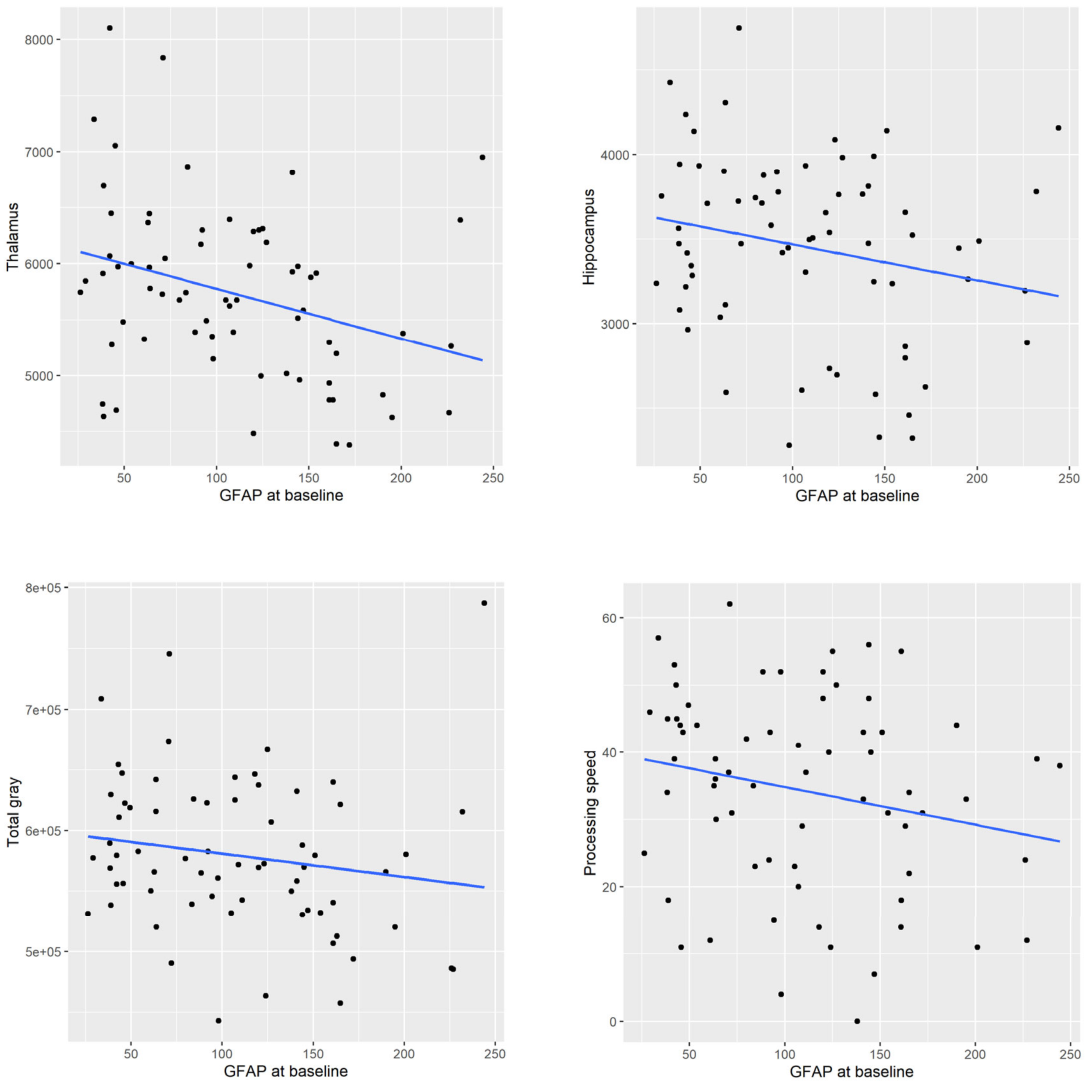

GFAP (measured in pg/mL) and MRI volume measurements (in mm^3), along with processing speed (computed from number of correct responses on Symbol Digit Coding test minus errors).
