# Supplementary figures and images for: Blood biomarkers and neurodegeneration in individuals exposed to repetitive head impacts
Source: Alzheimers Res Ther. 2023 Oct 12;15:173. doi: 10.1186/s13195-023-01310-w (PMC10571311; doi:10.1186/s13195-023-01310-w)

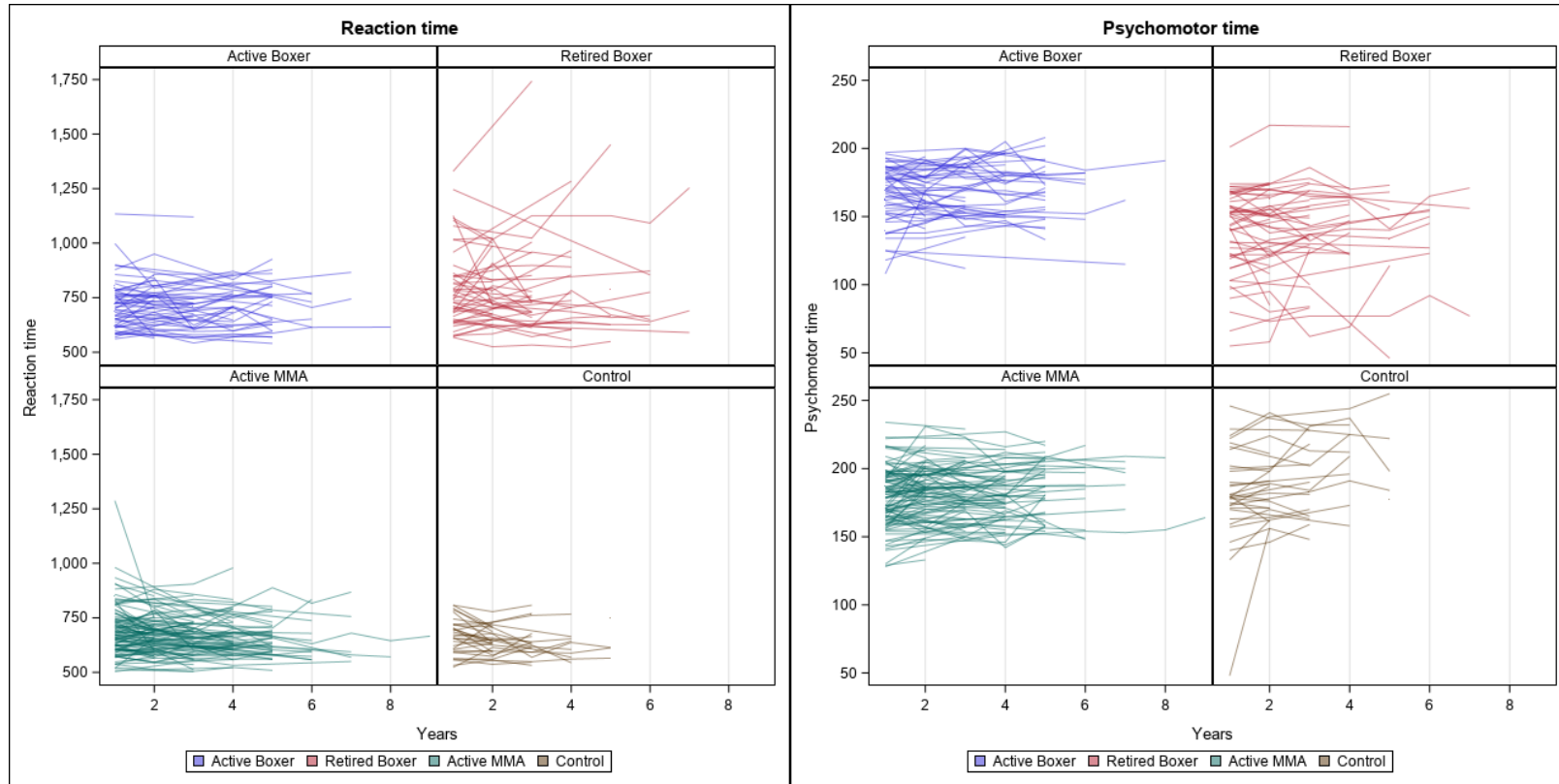

Supplement: Supplementary file 2 — Additional file 2: Fig. S4. Longitudinal measurement of reaction time ((Stroop Test Complex Reaction Time Correct + Stroop Reaction Time Correct)/2) and psychomotor speed [Finger Tap Test (FTT) Right Taps Average + FTT Left Taps Average + SDC Correct Responses) in active MMA fighters, active boxers, retired boxers, and control subjects) [file 13195_2023_1310_MOESM2_ESM.pdf]
